# Advancements in Cellular Therapeutics in Corneal Diseases

**DOI:** 10.3390/cells14231838

**Published:** 2025-11-21

**Authors:** Elizabeth Woo, Daniel Henick, Maryam Tahvildari

**Affiliations:** 1 Interdepartmental Neuroscience Program, Yale School of Medicine, New Haven, CT 06511, USA; 2 Department of Ophthalmology and Visual Science, Yale School of Medicine, New Haven, CT 06511, USA

**Keywords:** corneal epithelial stem cells, corneal endothelial cells, corneal stromal cells, mesenchymal stem cells, regenerative medicine, corneal blindness

## Abstract

Disorders of the cornea are responsible for a significant portion of preventable blindness worldwide. Various types of corneal transplantation procedures have successfully restored vision in many individuals; however, they carry the risk of graft failure due to immune rejection, endothelial cell dysfunction, infections and limbal stem cell deficiency. Thus, regenerative therapies of the cornea serve as promising alternatives or adjunct therapies. With improved understanding of limbal stem cell function and advancement in limbal stem cell culture technologies, major progress has been made in the in vivo and ex vivo cell-based therapies for treatment of corneal diseases. In this review, we summarize the recent developments achieved in cell-based therapeutics to target corneal epithelial, stromal, and endothelial cell disorders.

## 1. Introduction

According to the 2019 WHO report, approximately 2.2 billion people suffer from vision impairment worldwide [1]. While disorders of the cornea represent only one component of this burden, they also constitute a significant portion of preventable blindness, especially in lower-resourced countries [2]. For instance, corneal scarring remains the most common cause of blindness in young children in low-income countries [1]. While corneal graft tissue transplantation is a mainstay of treatment for many corneal diseases, it is limited by the shortage of viable corneal graft tissue. This limited access to tissue disproportionately impacts lower-income countries. In addition, corneal transplantation may not restore sight in eyes that have lost corneal clarity due to loss of corneal limbal stem cells (LSCs), secondary to inflammation (such as in Stevens–Johnson Syndrome, ocular cicatricial pemphigoid), severe infectious keratitis, and chemical, thermal, or mechanical injuries to the ocular surface.

Cell-based therapies, such as stem cell transplantation or genetically modified cells, have become a promising form of regenerative medicine. These advanced therapies aim to repair or replace the damaged organ. In addition to reducing reliance on tissue grafts, further optimization and implementation of these techniques could lead to procedures and protocols that may be more accessible in lower-resourced settings. This review will summarize the most recent advances in cell-based therapies targeting disorders of the cornea and discuss their limitations and challenges.

## 2. Corneal Anatomy

The cornea is the outermost, transparent layer of the eye. It comprises five distinctive layers (from exterior to interior): epithelium, Bowman’s membrane, stroma, Descemet’s membrane, and the endothelium. The role of the cornea is primarily two-fold, serving as a physical barrier against foreign particulates and providing 65% to 75% of total refraction power through the cornea’s curvature and thickness.

### 2.1. The Corneal Epithelium and the Bowman’s Membrane

The outermost surface of the cornea is a non-keratinizing, stratified squamous epithelium comprising five to seven layers of cells (superficial, wing, and basal cells). It serves as the main protective barrier to the outside environment and creates the smooth optical surface necessary for vision. The superficial cells of the epithelium are held together by desmosomes, preventing tears (as well as toxins and microbes) from entering the intercellular spaces. The superficial epithelium sloughs off regularly (7 to 10 days) and is constantly regenerated [3,4]. The basal cells comprise the deepest layer of the epithelium and are uniquely capable of mitosis, generating the superficial and wing cells of the cornea. The basal cells are attached to the basement membrane by hemidesmosomes. These cells are also responsible for secreting the Type IV collagen and laminin that maintain the basement membrane. Basal cells are disrupted in corneal erosions and nonhealing epithelial defects. The peripheral corneal epithelium, unlike central corneal epithelium, contains a reservoir of Langerhans cells, which are critical in surveillance of ocular antigens and ocular surface immunology. Thus, it serves as an important barrier.

Bowman’s membrane is an acellular membrane underneath the epithelium consisting of collagen types I and V as well as proteoglycans, rendering it critical for the maintenance of corneal structure. This structure can be fragmented or absent in keratoconus. Disruptions in the Bowman layer will often lead to corneal scarring.

### 2.2. The Corneal Stroma

The stroma also plays a critical role in maintaining the spherical shape of the cornea [5]. It is the thickest layer in the cornea and is made up of extracellular matrix (ECM), keratinocytes, and nerve fibers. It is maintained by keratocytes, which secrete matrix metalloproteinases (MMPs) and synthesize various collagen molecules and glycosaminoglycans.

### 2.3. Descemet’s Membrane and Corneal Endothelial Cell Layer

Descemet’s membrane is a 7 μm basement membrane layer composed largely of Type IV collagen and laminin. It is secreted by the endothelial cells that underlie it.

The endothelium is the innermost layer of the cornea. The honeycomb-like layer formed by hexagonal-shaped cells is highly metabolically active with two critical ion transport systems: membrane-bound Na^+^-K^+^ ATPase pumps and the intracellular carbonic anhydrase system [5]. These systems are critical for pumping water out of the cornea’s stroma and into the aqueous humor [6]. This function is critical for providing transparency and optical clarity to the cornea [7]. When endothelial cells are lost, the remaining cells migrate and enlarge to cover the empty space [8]. Unlike the epithelium, the endothelium does not proliferate in vivo, though they likely retain proliferative capacity [9]. As such, significant endothelial cell loss overwhelms the migratory capacity of the remaining endothelium which leads to visually significant corneal edema and breakdown of the corneal stroma and epithelium. This is frequently treated with endothelial keratoplasty to replace endothelial cell population.

**Figure 1:**
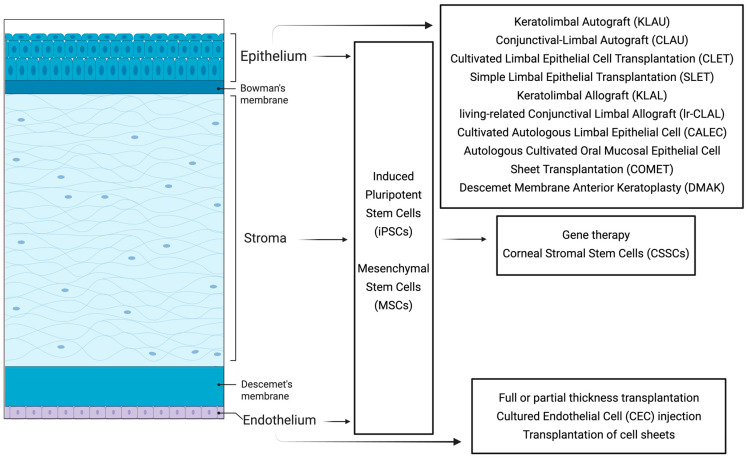
Stem cell-based therapies. Schema of the layers of the cornea (epithelium, Bowman’s membrane, stroma, Descemet’s membrane, and endothelium) with a summary of the cell-based therapeutics targeting disorders of the epithelium, stroma, and endothelium. iPSCs and MSCs are utilized to treat disorders of the three layers. Adapted from [5,7,10,11,12,13,14,15,16]. Created with BioRender.com.

Current cell-based therapies target disorders of the corneal epithelium, stroma, and endothelium, and each category will be discussed herein (summarized in Figure 1).

## 3. Corneal Epithelial Disorders

The current model governing epithelium homeostasis is the limbal epithelial stem cell (LESC) hypothesis which is based on the earlier XYZ hypothesis. Briefly, the XYZ hypothesis states that there is a migration of limbal stem cells centripetally (X), followed by vertical proliferation and differentiation of basal cells (Y), which is balanced by the shedding of squamous cells from the epithelial surface (Z) [17,18,19]. According to the LESC hypothesis, LESCs arise from the Palisades of Vogt at the limbus and divide centripetally to give rise to transient amplifying cells (TACs) in the basal epithelial layer. The TACs then proliferate, differentiate, and migrate vertically to the corneal surface where they terminally differentiate, after which they can shed and desquamate [19,20].

A competing model for epithelial homeostasis is the corneal epithelial stem cell (CESC) hypothesis [20,21]. This model posits that maintenance of the epithelium relies on stem cells that already inhabit the corneal basal layer rather than the LESCs in the peripheral corneal niches. According to this model, LESCs are only necessary under conditions of wound healing [21,22,23].

Limbal stem cell deficiency (LSCD) is a debilitating disorder characterized by either a loss or deficiency of the stem cells in the limbus. This impairs regeneration of corneal epithelium, leading to persistent epithelial defects, characteristic irregular “whorled” epithelium, corneal neovascularization, scarring, conjunctivalization, and inflammation. The etiology of LSCD is broad: genetic (PAX6 mutations, aniridia, Peter’s anomaly), inflammatory (Stevens–Johnson Syndrome, ocular cicatricial pemphigoid), infectious (severe bacterial, fungal or parasitic infections, herpetic keratopathy, trachoma), trauma (such as inappropriate contact lens use), chemical or thermal burns, iatrogenic, and ocular surface tumors. LSCD can present as either a unilateral or bilateral disease.

Management is initially symptom-directed, prioritizing optimization of the ocular surface with aggressive lubrication, and addressing the underlying cause for LSCD, which may maintain the remaining regenerative capacity of the ocular surface. However, with increasing disease severity, surgical management becomes necessary. Full thickness or partial thickness corneal transplants are not viable options as the donor tissue does not include limbal stem cells. In unilateral cases of LSCD, limbal stem cells can be harvested and transplanted from the other eye, but in bilateral or severe cases, allogeneic stem cell transplantation is required, which necessitates lifelong systemic immunosuppression. As such, LSCD has been a primary target for cell-based therapies.

Since the first published report of limbal stem cell transplantation in 1989 by Kenyon and Tseng, multiple stem cell-based therapy approaches have emerged. These therapies vary in terms of indication (unilateral vs. bilateral LSCD, primary pterygium) [24,25,26]: autologous vs. allogenic [27,28,29], tissue origins (limbal stem cells, induced pluripotent stem cells (iPSCs), mesenchymal stem cells, oral mucosa) [30,31,32,33], substrate (human amniotic membrane, fibrin, collagen, contact lenses, hydrogels) [34,35,36,37,38,39,40,41,42], feeder cells (mouse 3T3 fibroblasts, human dermal fibroblasts, human bone marrow-derived mesenchymal stem cells) [43,44], and the culture media used for tissue storage (fetal calf serum, autologous serum, serum-free) [45,46,47]. These are summarized in Table 1. We discuss each of these therapies, with ocular burns being the most common indication, in the sections below.

This table summarizes the main variables for stem cell-based therapies and specific examples for each variable.

### 3.1. Conjunctival–Limbal Autograft (CLAU) and Keratolimbal Autograft (KLAU)

Barraquer and Strampelli first proposed limbal autografts in 1964, which Kenyon and Tseng demonstrated as a reliable and viable treatment in 26 patients with a range of conditions, such as chemical or thermal injuries and contact lens-induced failure [48]. CLAU involves grafting a relatively large piece of conjunctiva and limbus retrieved from the patient’s other eye, while KLAU consists of the limbus and part of the peripheral cornea. As a result, both procedures necessitate a healthy, unaffected fellow eye. CLAU was the most used surgical approach for severe unilateral LSCD up until 2006 [49]. Longitudinal studies have shown that approximately 35–88% of eyes that underwent CLAU showed an improvement in the ocular surface and improved best-corrected visual acuity of two lines or more with minimal complications [50,51,52]. It is also used to treat primary pterygium and in a randomized controlled trial, CLAU was shown to result in minimal recurrence as compared to another technique called, mini-SLET (discussed in Section 3.3.) [53].

One of the benefits of CLAU and KLAU is the zero risk for immune rejection, which avoids long-term immunosuppression therapy. A concern with this technique is the potential for causing LSCD in the healthy donor eye, especially in those wearing contact lenses [54,55,56,57]. Though the true rate of iatrogenic LSCD is unknown [49], a large retrospective study that followed up on 45 patients who underwent CLAU alone, combined KLAU with CLAU, or living-related CLAL (conjunctival limbal allograft, discussed in Section 3.4) for a mean follow-up of 48.3 months found no signs of LSCD in the donor eye [58].

Since the introduction of CLAU and KLAU, there have been many modifications to the protocol. For instance, combination of CLAU with amniotic membrane transplantation has been shown to improve vision and maintain long-term ocular stability [26,34,59,60]. Recent work has further adapted this technique as the so-called sandwich (amnion/CLAU/amnion) transplantation, in the setting of recurrent pterygium and strabismus [61]. Another recent modification is the mini-CLAU technique (one to two clock hours vs. six to eight clock hours graft size) in combination with platelet-rich plasma (PRP) eye drops for treatment of unilateral total LSCD, to stabilize epithelialization [62,63]. This is built on prior work using amniotic membrane extract eye drops (AMEED) [64]. Unlike AMEED, PRP eye drops are derived from the patient’s blood, limiting risk of infectious transmission, decreasing cost, and simplifying preparation [62,63]. Long-term outcomes for such techniques are yet to be investigated.

### 3.2. Ex Vivo-Cultivated Limbal Epithelial Cell Transplantation (CLET)

Ex vivo-cultivated limbal epithelial cell transplantation was first introduced by Pellegrini in 1997, when human corneal epithelium was successfully cultivated in vitro and transplanted onto two patients [28]. CLET addressed two disadvantages of the classic CLAU technique: (1) risk of development of LSCD in the donor eye and (2) consumption of large amounts of tissue. CLET involves removing a much smaller graft area (2 mm^2^) from a healthy donor eye, culturing the biopsy for 2 weeks on a carrier scaffold, often human amniotic membrane (HAM), fibrin, or stem cell construct, before grafting onto the ocular surface [28,33,65,66,67]. In February 2015, the European Medicines Agency conditionally approved Holoclar in Europe [68]. The advantage of CLET is primarily that of using less donor tissue; however, it requires clean and equipped laboratory space to be able to cultivate ex vivo, which contributes to higher costs.

### 3.3. Simple Limbal Epithelial Transplantation (SLET)

In 2012, Sangwan et al. introduced the SLET approach, in which donor stem cells are directly seeded onto an amniotic membrane, which is then placed onto the ocular surface. An advantage of this approach is avoiding the additional steps of cellular expansion and using significantly less donor tissue [69]. Another advantage is cost-effectiveness, as it is about 10% that of culturing techniques, such as CLET [70]. SLET has been largely utilized in patients with unilateral chemical or thermal injuries and iatrogenic LSCD from multiple surgeries [10,69]. Though it is a relatively newer technique compared to CLAU and CLET, early outcome studies and, most recently, a prospective 5–12 years outcome study have consistently demonstrated SLET to be as effective as CLAU, and more so than CLET [10,27,71,72,73].

Though SLET has traditionally utilized amniotic membrane as a scaffold, recent work by Jain et al., has proposed SLET without amniotic membrane grafting in six patients with LSCD [74]. The authors performed limbal biopsy and using fibrin glue, directly secured the limbal explant onto the bare cornea [74]. Two other recent advances in SLET are mini-SLET and glueless-SLET (G-SLET) [75]. Mini-SLET has been utilized for treatment of LSCD in pediatric populations as well as pterygium treatment [24,76]. The mini-SLET technique involves a small limbal biopsy (of 2 mm in length) that is then cut into pieces and glued over an amniotic membrane. This technique has been especially beneficial in pediatric populations where the risk of dislodgement is greater [76]. Variations on the mini-SLET also include sandwiching the limbal explant between two amniotic membrane pieces [24,75]. To address unilateral LSCD where fibrin glue is not available, Malyugin proposed G-SLET as an acceptable alternative strategy, where limbal micrografts are harvested from the patient’s healthy eye and placed into small, self-sealing corneal tunnels in the affected eye [77]. This has been paired with low-energy femtosecond laser to standardize the corneal tunnel localizations and tunnel formation, which has shown stable epithelization in two patients within 2–3 weeks and improved best-corrected visual acuity (BCVA) [78,79]. Given the novelty of these techniques, the long-term outcomes remain to be seen.

In a systematic review comparing CLET with CLAU and SLET, the authors found the rate of anatomical and functional success to be lower in CLET as compared to CLAU and SLET at a follow-up of 1.75 years [27]. Importantly, the primary indication for transplantation in this review was ocular burns, which is one of many indications for transplantation. This suggests that careful consideration of the underlying pathology is necessary when choosing a therapeutic approach.

### 3.4. Keratolimbal Allograft (KLAL) and Living-Related Conjunctival Limbal Allograft (lr-CLAL)

In cases of a bilateral LSCD, an allogenic limbal stem cell transplantation (LSCT) would be the remaining choice. KLAL and lr-CLAL are considered for patients with bilateral LSCD without a related donor and patients with unilateral LSCD without a viable other eye [11]. KLAL is especially appropriate for patients with aniridia, contact lens wear-related LSCD, and iatrogenic LSCD that does not involve the conjunctiva. KLAL involves attaching limbal tissue to a corneoscleral carrier from a deceased donor. In contrast, lr-CLAL involves transplantation of limbal and conjunctival tissue with goblet cells from a matched living relative, thus making it ideal for patients with accompanying conjunctival disease.

According to a large retrospective cohort study assessing demographics and VA outcomes in patients who have undergone allogenic LSCT, patients across most age groups have a significant improvement in logMAR VA 12 months after LSCT (with the exception of those under 20 years old) [29]. Notably, patients with infectious cases and cases of unknown etiology demonstrated the least improvement at 12 months [29]. The disadvantages of allograft techniques are the risk of rejection, long-term immunosuppression, and limitations of donor tissue availability [65,80,81]. To address rejection, as part of lr-CLAL, groups have developed donor–recipient matching processes (Cincinnati donor selection protocol) to select the ideal donor with minimal antigenic burden [58]. For both KLAL and lr-CLAL, immunosuppression is critical for the longevity and stability of the graft. Immunosuppression includes oral tacrolimus, mycophenolate mofetil, prednisone, and antimicrobials. In the absence of randomized controlled studies, a review comparing outcomes and the literature of KLAL and lr-CLAL suggested a trend of lower rejection rate and graft failure with lr-CLAL [82,83,84].

### 3.5. Cultivated Autologous Limbal Epithelial Cell (CALEC)

One of the greatest challenges in gaining FDA approval for CLET protocols in the US has been developing a CLET protocol compliant with Good Manufacturing Practices (GMP). To address this, CALEC was recently developed which used only FDA-compliant materials, without allogeneic or xenogeneic feeder cells [14,85,86]. The authors demonstrated compelling feasibility, safety, and efficacy of data from a phase I/II trial of the first CLET protocol, in addition to it being xenobiotic-, serum-, and antibiotic-free [45,85]. Specifically, phase II trial results showed 92% of grafts had partial or complete success at 18 months without safety concerns [45]. This opens an important pathway for other LSC-based therapies in the US, allowing patients with LSCD to have access to regenerative eye-saving procedures.

### 3.6. Autologous Cultivated Oral Mucosal Epithelial Cell Sheet Transplantation (COMET)

For patients without any LSCs available for ex vivo expansion, such as in total bilateral LSCD, COMET is an alternative procedure. The first human transplantation of autologous oral mucosal cells cultured ex vivo on HAM was achieved in 2004, offering patients the alternative of using allogenic tissue and avoiding of immunosuppression in patients with severe ocular surface diseases, especially those caused by autoimmune diseases [87,88,89]. COMET encourages re-epithelialization and inhibits corneal melting and stabilizes the corneal surface in patients with acute corneal burns [90]. The advantage of COMET, in addition to minimizing risk of graft rejection, is that the procedure can be repeated if necessary.

A review of available clinical outcomes of COMET between 2004 and 2019 reported that COMET achieved stable ocular surface in 70.8% of eyes and improved visual acuity in 68.2% of eyes [30]. In 2021, the Japanese regulatory agency approved Ocural, the first product in the world for commercial COMET use in patients with LSCD. Early clinical outcomes from two patients who were treated with Ocural demonstrated successful engrafting [91]. In parallel, a prospective, randomized controlled clinical trial in India compared COMET and conjunctival-cultivated epithelial transplantation for patients with bilateral ocular surface disease secondary to SJS or chemical injuries [92]. The study showed that while both approaches did not offer long-term sustained BCVA improvements, COMET group patients had significantly better corneal clarity and improved corneal epithelialization at follow up [92]. An important negative sequela of COMET, however, is angiogenesis following transplantation [93]. In another study, COMET was found to be safe and effective for symblepharon release and long-term fornix reconstruction in eyes with chronic cicatrization [94].

### 3.7. Induced Pluripotent Stem Cells (iPSCs)

Human-iPSCs are derived from adult cells (e.g., skin or blood) and can be reprogrammed into corneal epithelial cells or corneal limbal stem cells [95,96,97,98]. Thus, they allow the generation of corneal epithelial stem cells and restore those niches in patients with LSCD, for instance, while avoiding potential ethical issues. Hayashi et al. first reported a method for extracting iPSCs from adult limbal epithelial cells and human dermal fibroblasts to generate corneal epithelial cells [99]. In 2016, Hayashi et al. further developed this method to generate a self-formed ectodermal autonomous multi-zone (SEAM) of ocular cells to better mimic whole eye development processes [95]. Using this technique, Soma et al. completed the first in human transplantation of iPSC-derived corneal epithelial cell sheets (iCEPSs) onto the ocular surface of four patients with LSCD [100]. The study found no clinical rejection or tumorigenesis (primary endpoint measure) for all four patients at 2-year follow up, and improvements in clinical staging of LSCD in half of the patients [100]. These findings are especially important, as a major concern for iPSC therapies is the risk of tumorigenicity. Though these results are promising, much larger clinical trials will be needed to demonstrate safety and efficacy.

### 3.8. Mesenchymal Stem Cells (MSCs)

MSCs can be harvested from various tissues such as bone marrow, adipose, and umbilical cord [96,101]. The MSCs are thought to make and secrete collagen, matrix metalloproteinases, growth factors, cytokines (e.g., TGF-β, IL-6), as well as extracellular vesicles (EVs), implicating them as important immunoregulatory agents [102,103,104,105,106]. This gives them a particular advantage for use in corneal allograft transplantation. For instance, in animal models of high-risk corneal transplantation, injection of subconjunctival MSC-derived exosomes or MSCs significantly prolonged graft survival time [107,108]. These characteristics make MSCs a potential therapeutic option for restoring defective LSC niches [109].

A proof-of-concept study demonstrated that bone marrow-derived MSC transplantation was equally as effective as allogenic CLET in improving central corneal epithelial phenotype at 12 months in patients with LSCD [110]. Another study showed that epithelial defects were absent in all eight patients with bilateral LSCD, who had received autologous adipose-derived MSCs even at 7 years, though only one patient maintained initial improvement [111]. A sub-analysis of a clinical trial assessing changes in limbal anatomy between MSC transplantation and CLET demonstrated that by in vivo confocal microscopy, both induced similar structural changes [112]. Furthermore, MSC-based therapy is safe, as analysis of studies using MSCs in more than 1000 patients did not demonstrate significant associations between MSC administration and tumor growth, death, infection, and toxicity [113]. However, there remain questions regarding the longevity of transplanted MSCs and their effects in vivo [114].

### 3.9. Descemet Membrane Anterior Keratoplasty (DMAK)

More recently, the regenerative properties of the Descemet membrane have been explored in its relation to healing the ocular surface in cases of LSCD [115]. Descemet membrane (DM) has been shown to be a viable culture substrate for ex vivo expansion of limbal stem cell populations. In this technique, a decellularized donor Descemet membrane is grafted onto a de-epithelialized corneal recipient bed. The cited case study combined this technique with allo-SLET from an ABO-matched cornea, as well as amniotic membrane. The ocular surface recovered with clear epithelium within 4 weeks, and vision had improved several lines with resolution of pain. There is limited research given the novelty of this technique, but further investigation may reveal a useful technique in regenerating the epithelium and limbal stem cell population.

The process of making these grafts is being further optimized and additional research is needed to demonstrate its utility in cases of LSCD. Further studies are also needed to determine which orientation of the decellularized DM graft optimizes recovery of the corneal epithelium.

## 4. Corneal Stromal Disorders

The 2015 International Committee for Classification of Corneal Dystrophies’ classification system re-classified stromal dystrophies as either epithelial-stromal or stromal dystrophies [116]. The epithelial-stromal corneal dystrophies are usually caused by mutations in transforming growth factor beta-induced gene, typically having an autosomal dominant inheritance, involving the Bowman layer and stroma. Some dystrophies under this category include Reis–Bucklers corneal dystrophy, Thiel–Behnke corneal dystrophy, lattice corneal dystrophy, and granular corneal dystrophies (and their variants). The stromal corneal dystrophies include macular corneal dystrophy, Schnyder corneal dystrophy, congenital stromal corneal dystrophy, posterior amorphous corneal dystrophy, central cloudy dystrophy of Francois, and pre-Descemet corneal dystrophy. Treatment options for stromal dystrophies typically include either phototherapeutic keratectomy (PTK), which uses an excimer laser to ablate the abnormal or damaged layers of the stroma, or corneal transplantation, such as penetrating keratoplasty or deep anterior lamellar keratoplasty (DALK) [117]. While the corneal stroma does not have the regenerative capacity of the epithelium, there is some potential for regeneration, and there have been efforts to harness stem cell technology, specifically mesenchymal stem cells. Unlike corneal epithelium, the corneal stroma is derived from a neural crest lineage. The complex structure of the corneal stroma has complicated efforts to create an effective corneal stroma construction. However, recent attention has been directed to the anti-inflammatory effect that corneal stromal cells may have on promoting a more natural environment for the proliferation of other cells and reducing scarring [118,119]. Gene therapy has also been of interest in treating certain types of corneal scarring.

In addition to corneal stromal dystrophies, which are largely driven by genetic mutations, stromal scarring (also known as fibrosis or late haze) can occur after injury, infection, or surgery to the cornea. For instance, photorefractive keratectomy (PRK), traumatic stromal injuries, chemical or thermal burns, microbial infections can all lead to stromal scarring [120]. The robust scarring of corneal stroma in injury is related to the loss of anti-fibrosis mediators and proliferation of extracellular matrix, with increased inflammatory signals leading to a wound-healing response [121]. Unlike other stem cell populations in the cornea, the corneal stromal stem cells not only need to self-renew and differentiate but also provide anti-fibrotic wound healing and modulation of the local immune environment to prevent scarring [122].

### 4.1. Corneal Stromal Stem Cells (CSSCs)

In addition to the more generalized category of mesenchymal stem cells, corneal stromal stem cells can be used to reduce corneal stromal scarring to prevent blindness. Though the in vitro behavior of corneal stromal stem cells (CSSCs) allows them to be characterized as MSCs, they are notably different. Both CSSC and MSC have the potential to differentiate into various cell types, but CSSCs differentiate into corneal keratocytes, which are responsible for the maintenance of corneal stroma, whereas the MSCs are a more broadly distributed population. In mouse models, direct injection of corneal stromal stem cells into the stroma has been shown to lead to successful differentiation to functional human stromal cells that express collagens and specific keratocyte markers with effective improvement in corneal clarity [123,124].

Pre-clinical studies of mouse models with acute stromal injury or alkali burns have demonstrated the potential of CSSCs in inhibiting corneal scarring and even restoring the uniformity of collagen fibrils [125,126,127,128]. For instance, Jhanji et al. found that CSSCs could be combined with corneal stromal keratocytes (CSKs) to potentiate regenerative capacity [128]. While CSSC provides modulation of the local immune system to prevent tissue inflammation and eventual fibrosis, CSK deposits specific collagens and other proteins to recover the stromal matrix needed for corneal clarity. In a mouse model, a combined CSSC and CSK treatment potentiated the effect of regenerating the corneal stroma and reducing corneal scarring. According to clinicaltrials.gov, there is an ongoing clinical trial evaluating the efficacy of corneal stromal lenticules with CSSCs and live keratocytes in treatment of stromal scars secondary to herpetic stromal keratitis.

Alió et al. conducted the first human clinical trial with patients suffering from advanced keratoconus, using both human autologous adipose-derived stem cells (ADASCs) and decellularized human corneal stroma to promote corneal healing [129]. Participants were randomized to receive ADASCs, corneal stroma, or a combination of the two. They found that at 3 months, there was recovery and full transparency in all groups and at 36 months, all cases had at least one to two lines of improvement in vision. On anterior segment optical coherence tomography, there was an increase in central corneal thickness and volume, though the effect was greater in patients who received decellularized corneal stroma. Flattening of the maximum keratometry readings was seen with all groups as well.

Exosomes produced by CSSCs are a particularly promising avenue of research, as they can promote cell proliferation, modulate inflammation, and remodel extracellular matrix [130]. Exosomes, extracellular vesicles (EVs) containing a variety of molecule types, are released by several cell types including corneal stromal cells. The utility of these exosomes in modulating the regenerative properties of stromal cells and regulating extracellular matrix remodeling and wound healing is currently being explored. One study showed that CSSC-derived exosomes containing miRNA could block corneal scarring in mice, thus there is potential to enhance the function of corneal stromal stem cells [131]. A recent study identified two anti-fibrotic miRNAs (miR-29a and 281-5p) derived from CSSC EVs, which could reduce corneal fibrosis in a mouse model of corneal stromal injury [132].

The possibility of rejuvenation of native corneal stromal stem cells is an area of active research, in which the use of specific mediators could enhance the inherent ability of corneal stromal stem cells to self-repair. Several growth factors, media, and gene therapy approaches are being explored to harness the stromal cell’s natural ability to replenish and heal [133] and also induce pluripotency of stem cells [134], while following Good Manufacturing Practices [135].

### 4.2. Gene Therapy

Though there are no currently available gene therapy strategies, there is great promise for gene therapy in targeting the inherited stromal dystrophies, as well as modulating corneal fibrosis, wound healing, and immune response. There are three current approaches to gene therapy for the treatment of inherited stromal dystrophies: (1) inactivation of the mutated gene, (2) gene correction or replacement with a normal copy, (3) addition of a healthy copy of the gene that will rescue the disease phenotype [15,136]. The viral vectors that have been tested in animal models include adeno-associated virus (AAV), lentivirus, and adenovirus. These vectors vary highly in terms of their ability to deliver and translate the desired protein [136].

However, several animal studies have shown promise in targeting corneal fibrosis. For instance, AAV5 has been successfully used to target TGF-ß signaling with Smad7 and Decorin in rabbit models of corneal scarring to reduce stromal haze and decrease corneal fibrosis [137,138,139]. Similarly, lentivirus-targeting Smad7 and p35 in mouse and rat models have shown to also decrease fibrosis markers and graft-mediated immune responses [140,141]. There have also been studies using adenovirus-targeting Smad7 and BMP-7 in mouse models of corneal scarring, demonstrating reduction in scarring or reduced activation of Smad3 signaling [142,143,144].

Gene therapy to prolong corneal allografts by transferring interleukin-10 and CTLA4-Ig has also been investigated [145,146]. Another study demonstrated that delivering a chimeric antivascularization immunomodulator using AAV8 ex vivo to corneal grafts showed reduction in inflammation and neovascularization [147].

Gene therapy will allow for more targeted, personalized therapeutic strategies. However, there remains much to be learned in terms of safety and efficacy.

## 5. Corneal Endothelial Disorders

The human corneal endothelium—unlike the epithelium—does not have regenerative capacity in vivo, though it has shown some capacity for proliferation in vitro [148,149]. Thus, in the setting of disease or injury, corneal endothelial cells (CECs) enlarge and migrate. Cell-based therapies are powerful tools to target endothelial disorders such as Fuchs endothelial corneal dystrophy, post-cataract extraction, and post-keratoplasty endothelial cell loss, or endothelial dysfunction in the setting of viral infections such as herpes simplex virus-associated endothelialitis.

### 5.1. Full Thickness or Partial Corneal Transplantation

Originally, the primary treatment for endothelial disorders was full-thickness penetrating keratoplasty. However, with the emergence of partial and endothelial corneal transplantation and Descemet membrane-stripping techniques, penetrating keratoplasty is no longer routinely used. Both full thickness and endothelial keratoplasties are relatively invasive strategies with risks of immunological rejection, infection, development of glaucoma, as well as limited donor tissue availability. In response to these limitations, other cell-based therapies have emerged, including direct injection of cultured cells into the anterior chamber, delivery of the cultured cells as cell sheets, and generation of iPSC-derived CECs.

### 5.2. Cultured Endothelial Cell (CEC) Injection Therapy

Several studies have successfully cultured CECs from both humans and nonhuman primates [9,150,151,152,153,154,155,156]. These earlier studies also reported the importance of Rho-associated coiled-coil-containing kinase (ROCK) inhibition, which is thought to promote engraftment of the CECs. Building on these studies, Kinoshita et al. first described the injection of cultured human CECs combined with an ROCK inhibitor into the anterior chamber of patients with bullous keratopathy [157]. The authors reported corneal restoration with normalized corneal thickness and resolution of corneal epithelial edema in all 11 patients who were followed for 2 years [157]. A five year follow up of these patients further demonstrated normal corneal endothelial function in 10 of the 11 eyes, reinforcing the long-term benefits of this strategy [158].

One of the limitations of the methodology used by Kinoshita et al. is that the distribution of the cells relies on gravity once injected, which can lead to heterogeneous distributions and continued proning. To address this, Bandeira et al. introduced injection of CECs embedded with magnetic nanoparticles into a rabbit corneal endothelial dystrophy model, which showed a more homogeneous distribution of CECs in the wound area when administered with nanoparticles [159]. A separate clinical trial in Mexico is testing the efficacy and safety of a magnetic nanoparticle-based CEC in patients with corneal edema.

These results offer yet other options for therapy for patients who would otherwise need to receive transplantation, but there remain challenges in allowing the therapy to be available in other countries.

### 5.3. Transplantation of Cell Sheets

Another mode of delivery of CECs has been the use of various scaffold materials. In this approach, the CECs are seeded onto a scaffold and then transplanted onto the cornea. The benefit to cell embedded grafts over injection of cells is that it not only replaces the diseased cells but replaces the underlying abnormal Descemet membrane. For instance, in the clinical trial of injected endothelial cells, deposits of guttae still existed on the DM, which had been left to promote cellular attachment. The challenges include creating a suitable cell carrier, such that it is transparent and stiff enough for surgical handling while also allowing good cellular adhesion. Prior studies have examined collagen, gelatin, human corneal stroma disk, and decellularized human corneal stroma disk, as well as a tissue-engineered cell sheet [151,160,161,162,163,164,165,166].

Tsai et al. tested for the first time the feasibility of tissue-engineered corneal endothelium transplantation, using a plastic-compressed collagen 3D matrix called Real Architecture for 3D Tissues (RAFTs) in an ex vivo human cornea organ culture system [167]. The authors seeded porcine cornea endothelial cells onto the RAFT and measured endothelial function even after two weeks [168]. Though this technology is in its nascency, it suggests that tissue-engineered grafts for transplantation may be a viable option for patients suffering from endothelial disorders.

### 5.4. Induced Pluripotent Stem Cell Therapy

Though CEC-based therapeutics are an exciting avenue of treatment for patients, they still require cadaveric donor corneas for CEC isolation and expansion. Thus, these therapeutics are limited by availability of donor tissue. As a result, a way to bypass this limitation is the use of iPSC-derived CECs. There have been several studies demonstrating the successful differentiation of CECs from iPSCs—either reprogramming adult somatic cells into iPSCs or directly differentiating them from established human iPSC lines [31,169]. In Japan, they have also created an HLA haplobank, where stocks of clinical grade iPSCs for HLA-matched allogenic transplantation are stored, as an alternative to generation of autologous iPSCs [170].

A first in human clinical trial demonstrated feasibility and safety of clinical-grade iPSC-derived CECs in treating bullous keratopathy in one patient, even after 1 year of follow-up [171]. The patient had modest improvement in corneal edema, transparency, and visual acuity [171]. Notably, the authors found de novo genetic mutations despite extensive whole-genome sequencing of the master cell bank, emphasizing the need for a more comprehensive safety strategy in iPSC-based therapies.

## 6. Future Directions and Conclusions

Numerous advancements have been made in the past decade in the treatment of blinding corneal diseases. Further understanding of the pathophysiology of limbal stem cell deficiencies, corneal scarring, and fibrosis, as well as corneal endothelial cell loss will lead to further individualized approaches in treatment options, improved outcomes, and reduced disease burden. This review has highlighted existing and newly developing cellular therapies to target endothelial, stromal, and epithelial disorders of the cornea. The next few years will likely see further development and refinement of the techniques described. In particular, the use of stem-cell-derived EVs, targeted gene therapy, organoids, 3D-printing of corneal layers, and ex vivo-modeling of stem cell niches will be promising avenues of research and potential clinical trials. Furthermore, the recent FDA approval of CALEC in the USA will certainly pave the way for other stem-cell therapies to be accessible to more patients.

Though it is clear that great progress has been made, there remain several challenges that range from safety and regulatory concerns to cost-effectiveness and accessibility. Safety concerns regarding tumorigenic behavior, unintended differentiation of cells, influence of the microenvironment on cellular differentiation, off-target effects of gene therapies have all been studied in limited time frames. As a result, more longitudinal follow-up and large clinical studies are necessary. Additionally, given the large variability in protocols for isolating, expanding, and generating cells, standardization across practices remains a critical need.

Finally, many of the discussed regenerative therapies require rigorous cell source isolation, testing, and transplantation, which limits accessibility in lower-resource countries and contributes to high costs. Thus, optimizing the manufacturing environment and protocols will be essential for popularizing these regenerative therapies.

## Figures and Tables

**Table 1:** Variables to consider for stem cell-based therapies.

Variables to Consider for Stem Cell-Based Therapies	
Indication	Unilateral vs. bilateral limbal stem cell deficiency, primary pterygium, recurrent pterygium
Autologous vs. allogenic	Autologous—from contralateral eye; Allogenic—from cadaveric tissue or living-related relative
Tissue source	Limbal stem cells, iPSCs, mesenchymal stem cells, oral mucosa
Substrate	Human amniotic membrane, fibrin, collagen, contact lenses, hydrogels
Feeder cells	Mouse 3T3 fibroblasts, human dermal fibroblasts, bone marrow-derived mesenchymal stem cells, xenobiotic free systems
Culture medium supplementation	Fetal calf serum, autologous serum, serum-free

## References

[1] World Health Organization World Report on Vision. https://www.who.int/publications/i/item/9789241516570.

[2] Burton M.J., Ramke J., Marques A.P., Bourne R.R.A., Congdon N., Jones I., Ah Tong B.A.M., Arunga S., Bachani D., Bascaran C. (2021). The Lancet Global Health Commission on Global Eye Health: Vision beyond 2020. Lancet Glob. Health.

[3] Wiley L., SundarRaj N., Sun T.T., Thoft R.A. (1991). Regional heterogeneity in human corneal and limbal epithelia: An immunohistochemical evaluation. Investig. Ophthalmol. Vis. Sci..

[4] Hanna C., Bicknell D.S., O’Brien J.E. (1961). Cell turnover in the adult human eye. Arch. Ophthalmol..

[5] DelMonte D.W., Kim T. (2011). Anatomy and physiology of the cornea. J. Cataract. Refract. Surg..

[6] Tuft S.J., Coster D.J. (1990). The corneal endothelium. Eye.

[7] Zavala J., Lopez Jaime G.R., Rodriguez Barrientos C.A., Valdez-Garcia J. (2013). Corneal endothelium: Developmental strategies for regeneration. Eye.

[8] Feizi S. (2018). Corneal endothelial cell dysfunction: Etiologies and management. Ther. Adv. Ophthalmol..

[9] Joyce N.C. (2012). Proliferative capacity of corneal endothelial cells. Exp. Eye Res..

[10] Basu S., Sureka S.P., Shanbhag S.S., Kethiri A.R., Singh V., Sangwan V.S. (2016). Simple Limbal Epithelial Transplantation: Long-Term Clinical Outcomes in 125 Cases of Unilateral Chronic Ocular Surface Burns. Ophthalmology.

[11] Cheung A.Y., Holland E.J. (2017). Keratolimbal allograft. Curr. Opin. Ophthalmol..

[12] Chen K., Soleimani M., Koganti R., Cheraqpour K., Habeel S., Djalilian A.R. (2023). Cell-based therapies for limbal stem cell deficiency: A literature review. Ann. Eye Sci..

[13] Daya S.M. (2017). Conjunctival-limbal autograft. Curr. Opin. Ophthalmol..

[14] Jurkunas U., Johns L., Armant M. (2022). Cultivated Autologous Limbal Epithelial Cell Transplantation: New Frontier in the Treatment of Limbal Stem Cell Deficiency. Am. J. Ophthalmol..

[15] Krajewska J.B., Waszczykowska A. (2025). Gene therapy strategies in ophthalmology-an overview of current developments and future prospects. J. Appl. Genet..

[16] Nuzzi A., Pozzo Giuffrida F., Luccarelli S., Nucci P. (2022). Corneal Epithelial Regeneration: Old and New Perspectives. Int. J. Mol. Sci..

[17] Thoft R.A., Friend J. (1983). The X, Y, Z hypothesis of corneal epithelial maintenance. Investig. Ophthalmol. Vis. Sci..

[18] Di Girolamo N. (2015). Moving epithelia: Tracking the fate of mammalian limbal epithelial stem cells. Prog. Retin. Eye Res..

[19] Dora N.J., Hill R.E., Collinson J.M., West J.D. (2015). Lineage tracing in the adult mouse corneal epithelium supports the limbal epithelial stem cell hypothesis with intermittent periods of stem cell quiescence. Stem Cell Res..

[20] Mort R.L., Douvaras P., Morley S.D., Dora N., Hill R.E., Collinson J.M., West J.D. (2012). Stem cells and corneal epithelial maintenance: Insights from the mouse and other animal models. Results Probl. Cell Differ..

[21] Majo F., Rochat A., Nicolas M., Jaoude G.A., Barrandon Y. (2008). Oligopotent stem cells are distributed throughout the mammalian ocular surface. Nature.

[22] Chang C.Y., Green C.R., McGhee C.N., Sherwin T. (2008). Acute wound healing in the human central corneal epithelium appears to be independent of limbal stem cell influence. Investig. Ophthalmol. Vis. Sci..

[23] Dua H.S., Miri A., Alomar T., Yeung A.M., Said D.G. (2009). The role of limbal stem cells in corneal epithelial maintenance: Testing the dogma. Ophthalmology.

[24] Hernandez-Bogantes E., Amescua G., Navas A., Garfias Y., Ramirez-Miranda A., Lichtinger A., Graue-Hernandez E.O. (2015). Minor ipsilateral simple limbal epithelial transplantation (mini-SLET) for pterygium treatment. Br. J. Ophthalmol..

[25] Daya S.M., Chan C.C., Holland E.J., on Behalf of the Members of The Cornea Society Ocular Surface Procedures Nomenclature Committee (2011). Cornea Society nomenclature for ocular surface rehabilitative procedures. Cornea.

[26] Yao T.Y., Wang J.S., Geng W., Xie H.T., Zhang M.C. (2023). Conjunctival Limbal Autograft Combined with Amnion-Assisted Conjunctival Epithelial Redirection for Unilateral Total Limbal Stem Cell Deficiency after Severe Chemical Burn. J. Clin. Med..

[27] Shanbhag S.S., Nikpoor N., Rao Donthineni P., Singh V., Chodosh J., Basu S. (2020). Autologous limbal stem cell transplantation: A systematic review of clinical outcomes with different surgical techniques. Br. J. Ophthalmol..

[28] Pellegrini G., Traverso C.E., Franzi A.T., Zingirian M., Cancedda R., De Luca M. (1997). Long-term restoration of damaged corneal surfaces with autologous cultivated corneal epithelium. Lancet.

[29] Tran J.A., Dohlman T.H., Zhang L.J., Lorch A., Elze T., Miller J.W., Yin J., Oke I., Dana R. (2025). Visual Outcomes of Limbal Stem Cell Transplantation in the IRIS(R) Registry. Ophthalmology.

[30] Cabral J.V., Jackson C.J., Utheim T.P., Jirsova K. (2020). Ex vivo cultivated oral mucosal epithelial cell transplantation for limbal stem cell deficiency: A review. Stem Cell Res. Ther..

[31] Ng X.Y., Peh G.S.L., Yam G.H., Tay H.G., Mehta J.S. (2023). Corneal Endothelial-like Cells Derived from Induced Pluripotent Stem Cells for Cell Therapy. Int. J. Mol. Sci..

[32] Surico P.L., Barone V., Singh R.B., Coassin M., Blanco T., Dohlman T.H., Basu S., Chauhan S.K., Dana R., Di Zazzo A. (2025). Potential applications of mesenchymal stem cells in ocular surface immune-mediated disorders. Surv. Ophthalmol..

[33] Burman S., Sangwan V. (2008). Cultivated limbal stem cell transplantation for ocular surface reconstruction. Clin. Ophthalmol..

[34] Meallet M.A., Espana E.M., Grueterich M., Ti S.E., Goto E., Tseng S.C. (2003). Amniotic membrane transplantation with conjunctival limbal autograft for total limbal stem cell deficiency. Ophthalmology.

[35] Pietryga K., Jesse K., Drzyzga R., Konka A., Zembala-John J., Kowalik A., Kielbowicz Z., Cwirko M., Buldak R.J., Dobrowolski D. (2024). Bio-printing method as a novel approach to obtain a fibrin scaffold settled by limbal epithelial cells for corneal regeneration. Sci. Rep..

[36] Ghezzi C.E., Rnjak-Kovacina J., Kaplan D.L. (2015). Corneal tissue engineering: Recent advances and future perspectives. Tissue Eng. Part. B Rev..

[37] Tidu A., Ghoubay-Benallaoua D., Lynch B., Haye B., Illoul C., Allain J.M., Borderie V.M., Mosser G. (2015). Development of human corneal epithelium on organized fibrillated transparent collagen matrices synthesized at high concentration. Acta Biomater..

[38] Polisetti N., Roschinski B., Schlotzer-Schrehardt U., Maier P., Schlunck G., Reinhard T. (2021). A Decellularized Human Limbal Scaffold for Limbal Stem Cell Niche Reconstruction. Int. J. Mol. Sci..

[39] Bobba S., Chow S., Watson S., Di Girolamo N. (2015). Clinical outcomes of xeno-free expansion and transplantation of autologous ocular surface epithelial stem cells via contact lens delivery: A prospective case series. Stem Cell Res. Ther..

[40] Gonzalez S., Chen L., Deng S.X. (2017). Comparative Study of Xenobiotic-Free Media for the Cultivation of Human Limbal Epithelial Stem/Progenitor Cells. Tissue Eng. Part C Methods.

[41] Brown K.D., Low S., Mariappan I., Abberton K.M., Short R., Zhang H., Maddileti S., Sangwan V., Steele D., Daniell M. (2014). Plasma polymer-coated contact lenses for the culture and transfer of corneal epithelial cells in the treatment of limbal stem cell deficiency. Tissue Eng. Part A.

[42] Rama P., Bonini S., Lambiase A., Golisano O., Paterna P., De Luca M., Pellegrini G. (2001). Autologous fibrin-cultured limbal stem cells permanently restore the corneal surface of patients with total limbal stem cell deficiency. Transplantation.

[43] Sharma S.M., Fuchsluger T., Ahmad S., Katikireddy K.R., Armant M., Dana R., Jurkunas U.V. (2012). Comparative analysis of human-derived feeder layers with 3T3 fibroblasts for the ex vivo expansion of human limbal and oral epithelium. Stem Cell Rev. Rep..

[44] Omoto M., Miyashita H., Shimmura S., Higa K., Kawakita T., Yoshida S., McGrogan M., Shimazaki J., Tsubota K. (2009). The use of human mesenchymal stem cell-derived feeder cells for the cultivation of transplantable epithelial sheets. Investig. Ophthalmol. Vis. Sci..

[45] Parekh M., Jurkunas U.V. (2025). Cultivated Autologous Limbal Epithelial Cell Transplantation: A Review of Clinical Trials and Applications. Cornea.

[46] Wu M.F., Stachon T., Seitz B., Langenbucher A., Szentmary N. (2017). Effect of human autologous serum and fetal bovine serum on human corneal epithelial cell viability, migration and proliferation in vitro. Int. J. Ophthalmol..

[47] Niruthisard D., Bonnet C., Tanasugarn L., Le B., Deng S.X. (2023). Autologous Serum Eye Drops in the Management of Limbal Stem Cell Deficiency Associated With Glaucoma Surgery. Eye Contact Lens.

[48] Kenyon K.R., Tseng S.C. (1989). Limbal autograft transplantation for ocular surface disorders. Ophthalmology.

[49] Le Q., Chauhan T., Yung M., Tseng C.H., Deng S.X. (2020). Outcomes of Limbal Stem Cell Transplant: A Meta-analysis. JAMA Ophthalmol..

[50] Eslani M., Cheung A.Y., Kurji K., Pierson K., Sarnicola E., Holland E.J. (2019). Long-term outcomes of conjunctival limbal autograft in patients with unilateral total limbal stem cell deficiency. Ocul. Surf..

[51] Kreimei M., Sorkin N., Einan-Lifshitz A., Rootman D.S., Chan C.C. (2019). Long-term outcomes of donor eyes after conjunctival limbal autograft and allograft harvesting. Can. J. Ophthalmol..

[52] Cauchi P.A., Ang G.S., Azuara-Blanco A., Burr J.M. (2008). A systematic literature review of surgical interventions for limbal stem cell deficiency in humans. Am. J. Ophthalmol..

[53] Oliva-Bienzobas V., Nava-Castaneda A., Jimenez-Corona A., Kahuam-Lopez N., Ramirez-Miranda A., Navas A., Graue-Hernandez E.O. (2023). Comparison of mini-simple limbal epithelial transplantation and conjunctival-limbal autograft for the treatment of primary pterygium: A randomised controlled trial. Br. J. Ophthalmol..

[54] Tan D.T., Ficker L.A., Buckley R.J. (1996). Limbal transplantation. Ophthalmology.

[55] Sangwan V.S., Basu S., Vemuganti G.K., Sejpal K., Subramaniam S.V., Bandyopadhyay S., Krishnaiah S., Gaddipati S., Tiwari S., Balasubramanian D. (2011). Clinical outcomes of xeno-free autologous cultivated limbal epithelial transplantation: A 10-year study. Br. J. Ophthalmol..

[56] Ramachandran C., Basu S., Sangwan V.S., Balasubramanian D. (2014). Concise review: The coming of age of stem cell treatment for corneal surface damage. Stem Cells Transl. Med..

[57] Rossen J., Amram A., Milani B., Park D., Harthan J., Joslin C., McMahon T., Djalilian A. (2016). Contact Lens-induced Limbal Stem Cell Deficiency. Ocul. Surf..

[58] Cheung A.Y., Sarnicola E., Kurji K.H., Govil A., Mogilishetty G., Eslani M., Wright E., Brailey P., Holland E.J. (2018). Cincinnati Protocol for Preoperative Screening and Donor Selection for Ocular Surface Stem Cell Transplantation. Cornea.

[59] Fallah M.R., Golabdar M.R., Amozadeh J., Zare M.A., Moghimi S., Fakhraee G. (2008). Transplantation of conjunctival limbal autograft and amniotic membrane vs mitomycin C and amniotic membrane in treatment of recurrent pterygium. Eye.

[60] Ruan Y., Jiang S., Musayeva A., Pfeiffer N., Gericke A. (2021). Corneal Epithelial Stem Cells-Physiology, Pathophysiology and Therapeutic Options. Cells.

[61] Wong H., Wang J.S., Du Y.L., Xie H.T., Zhang M.C. (2022). Sandwich (Amnion/Conjunctival-Limbal Autograft/Amnion) Transplantation for Recurrent Pterygium with Restrictive Strabismus. J. Clin. Med..

[62] Baradaran-Rafii A., Asl N.S., Ebrahimi M., Jabbehdari S., Bamdad S., Roshandel D., Eslani M., Momeni M. (2018). The role of amniotic membrane extract eye drop (AMEED) in in vivo cultivation of limbal stem cells. Ocul. Surf..

[63] Baradaran-Rafii A., Heidari-Keshel S., Behnaz N., Alemzadeh-Ansari M., Feizi S., Hassanpour K., Sadoughi M.M., Filutowski O., Ghahari M. (2023). Mini-Conjunctival Limbal Autograft (Mini-CLAU) Using Platelet-Rich Plasma Eye Drops (E-PRP): A Case Series. Cornea.

[64] Murri M.S., Moshirfar M., Birdsong O.C., Ronquillo Y.C., Ding Y., Hoopes P.C. (2018). Amniotic membrane extract and eye drops: A review of literature and clinical application. Clin. Ophthalmol..

[65] Ghareeb A.E., Lako M., Figueiredo F.C. (2020). Recent Advances in Stem Cell Therapy for Limbal Stem Cell Deficiency: A Narrative Review. Ophthalmol. Ther..

[66] Rama P., Matuska S., Paganoni G., Spinelli A., De Luca M., Pellegrini G. (2010). Limbal stem-cell therapy and long-term corneal regeneration. N. Engl. J. Med..

[67] Kolli S., Lako M., Figueiredo F., Mudhar H., Ahmad S. (2008). Loss of corneal epithelial stem cell properties in outgrowths from human limbal explants cultured on intact amniotic membrane. Regen. Med..

[68] Pellegrini G., Ardigo D., Milazzo G., Iotti G., Guatelli P., Pelosi D., De Luca M. (2018). Navigating Market Authorization: The Path Holoclar Took to Become the First Stem Cell Product Approved in the European Union. Stem Cells Transl. Med..

[69] Sangwan V.S., Basu S., MacNeil S., Balasubramanian D. (2012). Simple limbal epithelial transplantation (SLET): A novel surgical technique for the treatment of unilateral limbal stem cell deficiency. Br. J. Ophthalmol..

[70] Thokala P., Singh A., Singh V.K., Rathi V.M., Basu S., Singh V., MacNeil S., Sangwan V.S. (2022). Economic, clinical and social impact of simple limbal epithelial transplantation for limbal stem cell deficiency. Br. J. Ophthalmol..

[71] Shanbhag S.S., Kate A., Ganguly S., Jakati S., Deshmukh R., Basu S. (2025). Five- to Twelve-Year Outcomes of Autologous Simple Limbal Epithelial Transplantation: Long-Term Corneal Epithelial Imaging and Phenotypic Analysis. Am. J. Ophthalmol..

[72] Arora R., Dokania P., Manudhane A., Goyal J.L. (2017). Preliminary results from the comparison of simple limbal epithelial transplantation with conjunctival limbal autologous transplantation in severe unilateral chronic ocular burns. Indian J. Ophthalmol..

[73] Vazirani J., Ali M.H., Sharma N., Gupta N., Mittal V., Atallah M., Amescua G., Chowdhury T., Abdala-Figuerola A., Ramirez-Miranda A. (2016). Autologous simple limbal epithelial transplantation for unilateral limbal stem cell deficiency: Multicentre results. Br. J. Ophthalmol..

[74] Jain N., Mittal V., Sanandiya D. (2025). Outcomes of Simple Limbal Epithelial Transplantation Without Amniotic Membrane Grafting in Unilateral Limbal Stem Cell Deficiency: A Case Series of 6 Patients. Cornea.

[75] Garg A., Goel K., Gour A., Sapra M., Sangwan V.S., Tripathi R., Tiwari A. (2025). Unveiling the Molecular Mechanisms Underlying the Success of Simple Limbal Epithelial Transplantation (SLET). Cells.

[76] Pannu A., Sati A., Mishra S.K., Kumar S., Dhar S. (2021). Innovative technique of mini-simple limbal epithelial transplantation in pediatric patients. Indian J. Ophthalmol..

[77] Malyugin B.E., Gerasimov M.Y., Borzenok S.A. (2020). Glueless Simple Limbal Epithelial Transplantation: The Report of the First 2 Cases. Cornea.

[78] Malyugin B.E., Kalinnikova S.Y., Knyazer B., Gerasimov M.Y. (2024). Midterm Outcomes of Autologous Glueless Simple Limbal Epithelial Transplantation for Unilateral Limbal Stem Cell Deficiency. Cornea.

[79] Malyugin B., Kalinnikova S., Nefedova O., Gerasimov M., Muller F., Bernau W. (2025). Autologous Glueless Simple Limbal Epithelial Transplantation for Unilateral Stem Cell Deficiency Using Femtosecond Laser-Assisted Limbal Stem Cell Harvesting: The Report of the First 3 Clinical Cases. Cornea.

[80] Ilari L., Daya S.M. (2002). Long-term outcomes of keratolimbal allograft for the treatment of severe ocular surface disorders. Ophthalmology.

[81] Liang L., Sheha H., Tseng S.C. (2009). Long-term outcomes of keratolimbal allograft for total limbal stem cell deficiency using combined immunosuppressive agents and correction of ocular surface deficits. Arch. Ophthalmol..

[82] Li J.Y., Cortina M.S., Greiner M.A., Kuo A.N., Miller D.D., Shtein R.M., Veldman P.B., Yin J., Kim S.J., Shen J.F. (2024). Outcomes and Complications of Limbal Stem Cell Allograft Transplantation: A Report by the American Academy of Ophthalmology. Ophthalmology.

[83] Holland E.J. (1996). Epithelial transplantation for the management of severe ocular surface disease. Trans. Am. Ophthalmol. Soc..

[84] Holland E.J., Mogilishetty G., Skeens H.M., Hair D.B., Neff K.D., Biber J.M., Chan C.C. (2012). Systemic immunosuppression in ocular surface stem cell transplantation: Results of a 10-year experience. Cornea.

[85] Jurkunas U.V., Kaufman A.R., Yin J., Ayala A., Maguire M., Samarakoon L., Johns L.K., Parekh M., Li S., Gauthier A. (2025). Cultivated autologous limbal epithelial cell (CALEC) transplantation for limbal tem cell deficiency: A phase I/II clinical trial of the first xenobiotic-free, serum-free, antibiotic-free manufacturing protocol developed in the US. Nat. Commun..

[86] Jurkunas U.V., Yin J., Johns L.K., Li S., Negre H., Shaw K.L., Samarakoon L., Ayala A.R., Kheirkhah A., Katikireddy K. (2023). Cultivated autologous limbal epithelial cell (CALEC) transplantation: Development of manufacturing process and clinical evaluation of feasibility and safety. Sci. Adv..

[87] Nakamura T., Inatomi T., Sotozono C., Amemiya T., Kanamura N., Kinoshita S. (2004). Transplantation of cultivated autologous oral mucosal epithelial cells in patients with severe ocular surface disorders. Br. J. Ophthalmol..

[88] Sotozono C., Inatomi T., Nakamura T., Ueta M., Imai K., Fukuoka H., Komai S., Ishida G., Kitazawa K., Yokoi N. (2020). Oral Mucosal Epithelial Transplantation and Limbal-Rigid Contact Lens: A Therapeutic Modality for the Treatment of Severe Ocular Surface Disorders. Cornea.

[89] Venugopal R., Nagpal R., Mohanty S., Sen S., Kashyap S., Agarwal T., Maharana P.K., Vajpayee R.B., Sharma N. (2021). Outcomes of Cultivated Oral Mucosal Epithelial Transplantation in Eyes With Chronic Stevens-Johnson Syndrome Sequelae. Am. J. Ophthalmol..

[90] Ma D.H., Kuo M.T., Tsai Y.J., Chen H.C., Chen X.L., Wang S.F., Li L., Hsiao C.H., Lin K.K. (2009). Transplantation of cultivated oral mucosal epithelial cells for severe corneal burn. Eye.

[91] Toshida H., Kasahara T., Kiriyama M., Iwasaki Y., Sugita J., Ichikawa K., Ohta T., Miyahara K. (2023). Early Clinical Outcomes of the First Commercialized Human Autologous Ex Vivo Cultivated Oral Mucosal Epithelial Cell Transplantation for Limbal Stem Cell Deficiency: Two Case Reports and Literature Review. Int. J. Mol. Sci..

[92] Tandon R., Pandey P.K., Khan T.A., Das A.K., Kalaivani M., Majood M., Kashyap S., Sen S., Lomi N., Gupta N. (2024). Comparative evaluation of autologous tissue-engineered ocular and oral mucosal tissue grafts- a prospective randomized controlled trial. BMC Biotechnol..

[93] Chen H.C., Yeh L.K., Tsai Y.J., Lai C.H., Chen C.C., Lai J.Y., Sun C.C., Chang G., Hwang T.L., Chen J.K. (2012). Expression of angiogenesis-related factors in human corneas after cultivated oral mucosal epithelial transplantation. Investig. Ophthalmol. Vis. Sci..

[94] Komai S., Inatomi T., Nakamura T., Ueta M., Horiguchi G., Teramukai S., Kimura Y., Kagimura T., Fukushima M., Kinoshita S. (2022). Long-term outcome of cultivated oral mucosal epithelial transplantation for fornix reconstruction in chronic cicatrising diseases. Br. J. Ophthalmol..

[95] Hayashi R., Ishikawa Y., Sasamoto Y., Katori R., Nomura N., Ichikawa T., Araki S., Soma T., Kawasaki S., Sekiguchi K. (2016). Co-ordinated ocular development from human iPS cells and recovery of corneal function. Nature.

[96] Ahmad S., Figueiredo F., Lako M. (2006). Corneal epithelial stem cells: Characterization, culture and transplantation. Regen. Med..

[97] Brzeszczynska J., Samuel K., Greenhough S., Ramaesh K., Dhillon B., Hay D.C., Ross J.A. (2014). Differentiation and molecular profiling of human embryonic stem cell-derived corneal epithelial cells. Int. J. Mol. Med..

[98] Shalom-Feuerstein R., Serror L., De La Forest Divonne S., Petit I., Aberdam E., Camargo L., Damour O., Vigouroux C., Solomon A., Gaggioli C. (2012). Pluripotent stem cell model reveals essential roles for miR-450b-5p and miR-184 in embryonic corneal lineage specification. Stem Cells.

[99] Hayashi R., Ishikawa Y., Ito M., Kageyama T., Takashiba K., Fujioka T., Tsujikawa M., Miyoshi H., Yamato M., Nakamura Y. (2012). Generation of corneal epithelial cells from induced pluripotent stem cells derived from human dermal fibroblast and corneal limbal epithelium. PLoS ONE.

[100] Soma T., Oie Y., Takayanagi H., Matsubara S., Yamada T., Nomura M., Yoshinaga Y., Maruyama K., Watanabe A., Takashima K. (2024). Induced pluripotent stem-cell-derived corneal epithelium for transplant surgery: A single-arm, open-label, first-in-human interventional study in Japan. Lancet.

[101] Liu J., Yu F., Sun Y., Jiang B., Zhang W., Yang J., Xu G.T., Liang A., Liu S. (2015). Concise reviews: Characteristics and potential applications of human dental tissue-derived mesenchymal stem cells. Stem Cells.

[102] Bains K.K., Fukuoka H., Hammond G.M., Sotozono C., Quantock A.J. (2019). Recovering vision in corneal epithelial stem cell deficient eyes. Cont. Lens Anterior Eye.

[103] Ghannam S., Pene J., Moquet-Torcy G., Jorgensen C., Yssel H. (2010). Mesenchymal stem cells inhibit human Th17 cell differentiation and function and induce a T regulatory cell phenotype. J. Immunol..

[104] Holan V., Hermankova B., Bohacova P., Kossl J., Chudickova M., Hajkova M., Krulova M., Zajicova A., Javorkova E. (2016). Distinct Immunoregulatory Mechanisms in Mesenchymal Stem Cells: Role of the Cytokine Environment. Stem Cell Rev. Rep..

[105] Kossl J., Bohacova P., Hermankova B., Javorkova E., Zajicova A., Holan V. (2021). Antiapoptotic Properties of Mesenchymal Stem Cells in a Mouse Model of Corneal Inflammation. Stem Cells Dev..

[106] Lee M.J., Ko A.Y., Ko J.H., Lee H.J., Kim M.K., Wee W.R., Khwarg S.I., Oh J.Y. (2015). Mesenchymal stem/stromal cells protect the ocular surface by suppressing inflammation in an experimental dry eye. Mol. Ther..

[107] Jia Z., Li F., Zeng X., Lv Y., Zhao S. (2018). The effects of local administration of mesenchymal stem cells on rat corneal allograft rejection. BMC Ophthalmol..

[108] Jia Z., Lv Y., Zhang W., Zhang X., Li F., Lu X., Zhao S. (2022). Mesenchymal stem cell derived exosomes-based immunological signature in a rat model of corneal allograft rejection therapy. Front. Biosci. (Landmark Ed.).

[109] Bonnet C., Gonzalez S., Deng S.X. (2024). Limbal stem cell therapy. Curr. Opin. Ophthalmol..

[110] Calonge M., Perez I., Galindo S., Nieto-Miguel T., Lopez-Paniagua M., Fernandez I., Alberca M., Garcia-Sancho J., Sanchez A., Herreras J.M. (2019). A proof-of-concept clinical trial using mesenchymal stem cells for the treatment of corneal epithelial stem cell deficiency. Transl. Res..

[111] Boto de Los Bueis A., Vidal Arranz C., Del Hierro-Zarzuelo A., Diaz Valle D., Mendez Fernandez R., Gabarron Hermosilla M.I., Benitez Del Castillo J.M., Garcia-Arranz M. (2024). Long-Term Effects of Adipose-Derived Stem Cells for the Treatment of Bilateral Limbal Stem Cell Deficiency. Curr. Eye Res..

[112] Perez I., Galindo S., Lopez-Miguel A., Nieto-Miguel T., de la Mata A., Lopez-Paniagua M., Alberca M., Herreras J.M., Calonge M. (2023). In Vivo Confocal Microscopy in Limbal Stem Cell Deficiency After Mesenchymal Stem Cell Transplantation: A Sub-analysis from a Phase I-II Clinical Trial. Ophthalmol. Ther..

[113] Lalu M.M., McIntyre L., Pugliese C., Fergusson D., Winston B.W., Marshall J.C., Granton J., Stewart D.J., Canadian Critical Care Trials Group (2012). Safety of cell therapy with mesenchymal stromal cells (SafeCell): A systematic review and meta-analysis of clinical trials. PLoS ONE.

[114] Venkatakrishnan J., Saeed Y., Kao W.W. (2022). Trends in using mesenchymal stromal/stem cells (MSCs) in treating corneal diseases. Ocul. Surf..

[115] Cheung A.Y., Reinisch C.B., Hou J.H. (2025). Decellularized Descemet Membrane Anterior Keratoplasty With Allogeneic Simple Limbal Epithelial Transplantation for Partial Limbal Stem Cell Deficiency Following Partial Keratolimbal Allograft Failure. Cornea.

[116] Weiss J.S., Moller H.U., Aldave A.J., Seitz B., Bredrup C., Kivela T., Munier F.L., Rapuano C.J., Nischal K.K., Kim E.K. (2015). IC3D classification of corneal dystrophies--edition 2. Cornea.

[117] Ashena Z., Niestrata M., Tavassoli S. (2023). Management of Stromal Corneal Dystrophies; Review of the Literature with a Focus on Phototherapeutic Keratectomy and Keratoplasty. Vision.

[118] El Zarif M., Alio Del Barrio J.L., Arnalich-Montiel F., De Miguel M.P., Makdissy N., Alio J.L. (2020). Corneal Stroma Regeneration: New Approach for the Treatment of Cornea Disease. Asia Pac. J. Ophthalmol..

[119] Suanno G., Genna V.G., Maurizi E., Dieh A.A., Griffith M., Ferrari G. (2024). Cell therapy in the cornea: The emerging role of microenvironment. Prog. Retin. Eye Res..

[120] Medeiros C.S., Marino G.K., Santhiago M.R., Wilson S.E. (2018). The Corneal Basement Membranes and Stromal Fibrosis. Investig. Ophthalmol. Vis. Sci..

[121] Volatier T., Cursiefen C., Notara M. (2024). Current Advances in Corneal Stromal Stem Cell Biology and Therapeutic Applications. Cells.

[122] Wang Y., Chen X., Cao W., Shi Y. (2014). Plasticity of mesenchymal stem cells in immunomodulation: Pathological and therapeutic implications. Nat. Immunol..

[123] Du Y., Carlson E.C., Funderburgh M.L., Birk D.E., Pearlman E., Guo N., Kao W.W., Funderburgh J.L. (2009). Stem cell therapy restores transparency to defective murine corneas. Stem Cells.

[124] Harkin D.G., Foyn L., Bray L.J., Sutherland A.J., Li F.J., Cronin B.G. (2015). Concise reviews: Can mesenchymal stromal cells differentiate into corneal cells? A systematic review of published data. Stem Cells.

[125] Khandaker I., Funderburgh J.L., Geary M.L., Funderburgh M.L., Jhanji V., Du Y., Hin-Fai Yam G. (2020). A novel transgenic mouse model for corneal scar visualization. Exp. Eye Res..

[126] Ghoubay D., Borderie M., Grieve K., Martos R., Bocheux R., Nguyen T.M., Callard P., Chedotal A., Borderie V.M. (2020). Corneal stromal stem cells restore transparency after N(2) injury in mice. Stem Cells Transl. Med..

[127] Basu S., Hertsenberg A.J., Funderburgh M.L., Burrow M.K., Mann M.M., Du Y., Lathrop K.L., Syed-Picard F.N., Adams S.M., Birk D.E. (2014). Human limbal biopsy-derived stromal stem cells prevent corneal scarring. Sci. Transl. Med..

[128] Jhanji V., Santra M., Riau A.K., Geary M.L., Yang T., Rubin E., Yusoff N., Dhaliwal D.K., Mehta J.S., Yam G.H. (2022). Combined Therapy Using Human Corneal Stromal Stem Cells and Quiescent Keratocytes to Prevent Corneal Scarring after Injury. Int. J. Mol. Sci..

[129] Alio Del Barrio J.L., El Zarif M., de Miguel M.P., Azaar A., Makdissy N., Harb W., El Achkar I., Arnalich-Montiel F., Alio J.L. (2017). Cellular Therapy With Human Autologous Adipose-Derived Adult Stem Cells for Advanced Keratoconus. Cornea.

[130] Samaeekia R., Rabiee B., Putra I., Shen X., Park Y.J., Hematti P., Eslani M., Djalilian A.R. (2018). Effect of Human Corneal Mesenchymal Stromal Cell-derived Exosomes on Corneal Epithelial Wound Healing. Investig. Ophthalmol. Vis. Sci..

[131] Shojaati G., Khandaker I., Funderburgh M.L., Mann M.M., Basu R., Stolz D.B., Geary M.L., Dos Santos A., Deng S.X., Funderburgh J.L. (2019). Mesenchymal Stem Cells Reduce Corneal Fibrosis and Inflammation via Extracellular Vesicle-Mediated Delivery of miRNA. Stem Cells Transl. Med..

[132] Yam G.H., Yang T., Geary M.L., Santra M., Funderburgh M., Rubin E., Du Y., Sahel J.A., Jhanji V., Funderburgh J.L. (2023). Human corneal stromal stem cells express anti-fibrotic microRNA-29a and 381-5p—A robust cell selection tool for stem cell therapy of corneal scarring. J. Adv. Res..

[133] Lane S.W., Williams D.A., Watt F.M. (2014). Modulating the stem cell niche for tissue regeneration. Nat. Biotechnol..

[134] Takahashi K., Yamanaka S. (2006). Induction of pluripotent stem cells from mouse embryonic and adult fibroblast cultures by defined factors. Cell.

[135] Santra M., Geary M.L., Rubin E., Hsu M.Y.S., Funderburgh M.L., Chandran C., Du Y., Dhaliwal D.K., Jhanji V., Yam G.H. (2024). Good manufacturing practice production of human corneal limbus-derived stromal stem cells and in vitro quality screening for therapeutic inhibition of corneal scarring. Stem Cell Res. Ther..

[136] Sarkar S., Panikker P., D’Souza S., Shetty R., Mohan R.R., Ghosh A. (2023). Corneal Regeneration Using Gene Therapy Approaches. Cells.

[137] He X., Fu Y., Ma L., Yao Y., Ge S., Yang Z., Fan X. (2023). AAV for Gene Therapy in Ocular Diseases: Progress and Prospects. Research.

[138] Gupta S., Rodier J.T., Sharma A., Giuliano E.A., Sinha P.R., Hesemann N.P., Ghosh A., Mohan R.R. (2017). Targeted AAV5-Smad7 gene therapy inhibits corneal scarring in vivo. PLoS ONE.

[139] Mohan R.R., Gupta S., Kumar R., Sinha N.R., Landreneau J., Sinha P.R., Tandon A., Chaurasia S.S., Hesemann N.P. (2024). Tissue-targeted and localized AAV5-DCN and AAV5-PEDF combination gene therapy abrogates corneal fibrosis and concurrent neovascularization in rabbit eyes in vivo. Ocul. Surf..

[140] Wang T., Zhou X.T., Yu Y., Zhu J.Y., Dai J.H., Qu X.M., Le Q.H., Chu R.Y. (2013). Inhibition of corneal fibrosis by Smad7 in rats after photorefractive keratectomy. Chin. Med. J..

[141] Pastak M., Kleff V., Saban D.R., Czugala M., Steuhl K.P., Ergun S., Singer B.B., Fuchsluger T.A. (2018). Gene Therapy for Modulation of T-Cell-Mediated Immune Response Provoked by Corneal Transplantation. Hum. Gene Ther..

[142] Nguyen P., Yiu S.C. (2013). Strategies for local gene therapy of corneal allograft rejection. Middle East. Afr. J. Ophthalmol..

[143] Saika S., Ikeda K., Yamanaka O., Miyamoto T., Ohnishi Y., Sato M., Muragaki Y., Ooshima A., Nakajima Y., Kao W.W. (2005). Expression of Smad7 in mouse eyes accelerates healing of corneal tissue after exposure to alkali. Am. J. Pathol..

[144] Saika S., Ikeda K., Yamanaka O., Flanders K.C., Nakajima Y., Miyamoto T., Ohnishi Y., Kao W.W., Muragaki Y., Ooshima A. (2005). Therapeutic effects of adenoviral gene transfer of bone morphogenic protein-7 on a corneal alkali injury model in mice. Lab. Investig..

[145] Parker D.G., Coster D.J., Brereton H.M., Hart P.H., Koldej R., Anson D.S., Williams K.A. (2010). Lentivirus-mediated gene transfer of interleukin 10 to the ovine and human cornea. Clin. Exp. Ophthalmol..

[146] Amador C., Shah R., Ghiam S., Kramerov A.A., Ljubimov A.V. (2022). Gene Therapy in the Anterior Eye Segment. Curr. Gene Ther..

[147] Gilger B.C., Hasegawa T., Sutton R.B., Bower J.J., Li C., Hirsch M.L. (2024). A chimeric anti-vascularization immunomodulator prevents high-risk corneal transplantation rejection via ex vivo gene therapy. Mol. Ther..

[148] Murphy C., Alvarado J., Juster R., Maglio M. (1984). Prenatal and postnatal cellularity of the human corneal endothelium. A quantitative histologic study. Investig. Ophthalmol. Vis. Sci..

[149] Edelhauser H.F. (2000). The resiliency of the corneal endothelium to refractive and intraocular surgery. Cornea.

[150] Mimura T., Yamagami S., Amano S. (2013). Corneal endothelial regeneration and tissue engineering. Prog. Retin. Eye Res..

[151] Mimura T., Yamagami S., Yokoo S., Usui T., Tanaka K., Hattori S., Irie S., Miyata K., Araie M., Amano S. (2004). Cultured human corneal endothelial cell transplantation with a collagen sheet in a rabbit model. Investig. Ophthalmol. Vis. Sci..

[152] Schmedt T., Silva M.M., Ziaei A., Jurkunas U. (2012). Molecular bases of corneal endothelial dystrophies. Exp. Eye Res..

[153] Eveleth D., Pizzuto S., Weant J., Jenkins-Eveleth J., Bradshaw R.A. (2020). Proliferation of Human Corneal Endothelia in Organ Culture Stimulated by Wounding and the Engineered Human Fibroblast Growth Factor 1 Derivative TTHX1114. J. Ocul. Pharmacol. Ther..

[154] Bartakova A., Kuzmenko O., Alvarez-Delfin K., Kunzevitzky N.J., Goldberg J.L. (2018). A Cell Culture Approach to Optimized Human Corneal Endothelial Cell Function. Investig. Ophthalmol. Vis. Sci..

[155] Engelmann K., Friedl P. (1995). Growth of human corneal endothelial cells in a serum-reduced medium. Cornea.

[156] Zhu C., Joyce N.C. (2004). Proliferative response of corneal endothelial cells from young and older donors. Investig. Ophthalmol. Vis. Sci..

[157] Kinoshita S., Koizumi N., Ueno M., Okumura N., Imai K., Tanaka H., Yamamoto Y., Nakamura T., Inatomi T., Bush J. (2018). Injection of Cultured Cells with a ROCK Inhibitor for Bullous Keratopathy. N. Engl. J. Med..

[158] Numa K., Imai K., Ueno M., Kitazawa K., Tanaka H., Bush J.D., Teramukai S., Okumura N., Koizumi N., Hamuro J. (2021). Five-Year Follow-up of First 11 Patients Undergoing Injection of Cultured Corneal Endothelial Cells for Corneal Endothelial Failure. Ophthalmology.

[159] Bandeira F., Grottone G.T., Covre J.L., Cristovam P.C., Loureiro R.R., Pinheiro F.I., Casaroli-Marano R.P., Donato W., Gomes J.A.P. (2023). A Framework for Human Corneal Endothelial Cell Culture and Preliminary Wound Model Experiments with a New Cell Tracking Approach. Int. J. Mol. Sci..

[160] Yamaguchi M., Shima N., Kimoto M., Ebihara N., Murakami A., Yamagami S. (2016). Optimization of Cultured Human Corneal Endothelial Cell Sheet Transplantation and Post-Operative Sheet Evaluation in a Rabbit Model. Curr. Eye Res..

[161] Lai J.Y., Chen K.H., Hsiue G.H. (2007). Tissue-engineered human corneal endothelial cell sheet transplantation in a rabbit model using functional biomaterials. Transplantation.

[162] Niu G., Choi J.S., Wang Z., Skardal A., Giegengack M., Soker S. (2014). Heparin-modified gelatin scaffolds for human corneal endothelial cell transplantation. Biomaterials.

[163] Honda N., Mimura T., Usui T., Amano S. (2009). Descemet stripping automated endothelial keratoplasty using cultured corneal endothelial cells in a rabbit model. Arch. Ophthalmol..

[164] Peh G.S.L., Ang H.P., Lwin C.N., Adnan K., George B.L., Seah X.Y., Lin S.J., Bhogal M., Liu Y.C., Tan D.T. (2017). Regulatory Compliant Tissue-Engineered Human Corneal Endothelial Grafts Restore Corneal Function of Rabbits with Bullous Keratopathy. Sci. Rep..

[165] Peh G.S.L., Ong H.S., Adnan K., Ang H.P., Lwin C.N., Seah X.Y., Lin S.J., Mehta J.S. (2019). Functional Evaluation of Two Corneal Endothelial Cell-Based Therapies: Tissue-Engineered Construct and Cell Injection. Sci. Rep..

[166] Levis H.J., Kureshi A.K., Massie I., Morgan L., Vernon A.J., Daniels J.T. (2015). Tissue Engineering the Cornea: The Evolution of RAFT. J. Funct. Biomater..

[167] Tsai R.J., Li L., Chen J. (2000). Reconstruction of damaged corneas by transplantation of autologous limbal epithelial cells(1). Am. J. Ophthalmol..

[168] Tsai M.C., Kureshi A., Daniels J.T. (2025). Tissue engineered corneal endothelium transplantation in an ex vivo human cornea organ culture model. Sci. Rep..

[169] Hatou S., Shimmura S. (2019). Review: Corneal endothelial cell derivation methods from ES/iPS cells. Inflamm. Regen..

[170] Yoshida S., Kato T.M., Sato Y., Umekage M., Ichisaka T., Tsukahara M., Takasu N., Yamanaka S. (2023). A clinical-grade HLA haplobank of human induced pluripotent stem cells matching approximately 40% of the Japanese population. Med.

[171] Hirayama M., Hatou S., Nomura M., Hokama R., Hirayama O.I., Inagaki E., Aso K., Sayano T., Dohi H., Hanatani T. (2025). A first-in-human clinical study of an allogenic iPSC-derived corneal endothelial cell substitute transplantation for bullous keratopathy. Cell Rep. Med..

